# Crystal Structure of the N-Acetylmannosamine Kinase Domain of GNE

**DOI:** 10.1371/journal.pone.0007165

**Published:** 2009-10-20

**Authors:** Yufeng Tong, Wolfram Tempel, Lyudmila Nedyalkova, Farrell MacKenzie, Hee-Won Park

**Affiliations:** 1 Structural Genomics Consortium, University of Toronto, Toronto, Ontario, Canada; 2 Department of Pharmacology, University of Toronto, Toronto, Ontario, Canada; University of Cambridge, United Kingdom

## Abstract

**Background:**

UDP-GlcNAc 2-epimerase/ManNAc 6-kinase, GNE, is a bi-functional enzyme that plays a key role in sialic acid biosynthesis. Mutations of the GNE protein cause sialurea or autosomal recessive inclusion body myopathy/Nonaka myopathy. GNE is the only human protein that contains a kinase domain belonging to the ROK (repressor, ORF, kinase) family.

**Principal Findings:**

We solved the structure of the GNE kinase domain in the ligand-free state. The protein exists predominantly as a dimer in solution, with small populations of monomer and higher-order oligomer in equilibrium with the dimer. Crystal packing analysis reveals the existence of a crystallographic hexamer, and that the kinase domain dimerizes through the C-lobe subdomain. Mapping of disease-related missense mutations onto the kinase domain structure revealed that the mutation sites could be classified into four different groups based on the location – dimer interface, interlobar helices, protein surface, or within other secondary structural elements.

**Conclusions:**

The crystal structure of the kinase domain of GNE provides a structural basis for understanding disease-causing mutations and a model of hexameric wild type full length enzyme.

**Enhanced Version:**

**This article can also be viewed as an enhanced version in which the text of the article is integrated with interactive 3D representations and animated transitions. Please note that a web plugin is required to access this enhanced functionality. Instructions for the installation and use of the web plugin are available in Text S1.**

## Introduction

**Figure 1:**
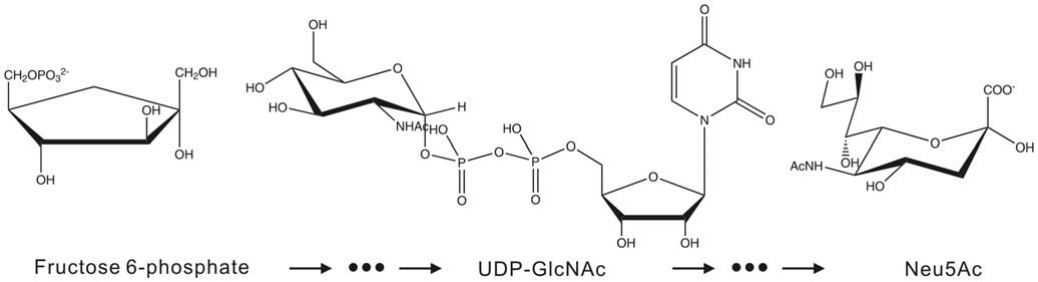
Key sugar molecules in the sialic acid biosynthesis pathway.

Sialic acids are N- or *O-* substituted terminal monosaccharides with a nine-carbon backbone highly expressed on eukaryotic cell surfaces [1]. Sialylation of glycoproteins and glycolipids modulates a wide range of biological and pathological events including early development [2], tumorigenesis [3], viral and bacterial infection, and immunity [4], [5]. In vertebrate systems, N-acetylneuraminic acid (Neu5Ac) is the metabolic precursor of all known naturally occurring sialic acids [6]. Neu5Ac is synthesized in the cytosol from UDP-N-acetylglucosamine (UDP-GlcNAc) by four consecutive reactions; and UDP-GlcNAc is a derivative of fructose-6-phosphate and the end-product of the hexosamine biosynthesis pathway (Figure 1).

The first two steps of the biosynthesis of Neu5Ac from UDP-GlcNAc are catalyzed by the bi-functional enzyme UDP-GlcNAc 2-epimerase/N-acetylmannosamine kinase (GNE). GNE contains an N-terminal epimerase domain and a C-terminal kinase domain [7]. The epimerase domain converts UDP-GlcNAc to N-acetylmannosamine (ManNAc), which is then phosphorylated at the 6 position by the kinase domain. GNE is feedback-inhibited by the activated form of Neu5Ac, i.e., cytidine-monophosphate N-acetylneuraminic acid (CMP-Neu5Ac). The kinase domain belongs to the ROK (Repressor, ORF, Kinase) family. The ROK family consists of a set of bacterial proteins that include repressors for sugar catabolic operons, and sugar kinases [8]. *Gne* is the only known gene in the entire human genome that encodes a ROK domain-containing protein.

Three protein isoforms have been described for human GNE, where isoform 1 is ubiquitously expressed and is believed to be responsible for the basic supply of sialic acids. Isoforms 2 and 3 are generated by alternative splicing and show tissue specific expression patterns. Isoforms 2 and 3 have reduced epimerase activities but almost intact kinase activities and may fine-tune the production of sialic acids [9]. Wild type GNE forms homo-hexamer in solution [10], and allosteric regulation of the epimerase and kinase activities of GNE is important for the normal function of the protein [10], [11]. Mutations in the epimerase domain lead to the rare congenital metabolism disorder sialurea, which results in the production of high levels of Neu5Ac due to loss of the allosteric feedback control of the UDP-GlcNAc 2-epimerase activity by CMP-Neu5Ac [12]. Late onset autosomal recessive inclusion body myopathy, which is also known as hereditary inclusion body myopathy (hereinafter referred to as HIBM), and allelic Nonaka myopathy are neuromuscular disorders that are caused by a number of different mutations within the *gne* gene. The mutations are located at either the epimerase domain or the kinase domain [13] and lead to hypoactivity of the enzyme [11]. Mutagenesis and enzymatic activity analysis revealed that the activities of the epimerase domain and the kinase domain are interrelated such that a single mutation in one domain could affect the activities of both domains [11]. Here, we solved the structure of the dimeric GNE kinase domain in the ligand-free state. The structure reveals the dimerization interface of the kinase domain and also suggests a possible hexameric assembly of the protein. Furthermore, the structure provides insights into the relationship between GNE mutations and GNE-related metabolism disorders.

## Results and Discussion

### Overview of the GNE kinase domain monomer

**Figure 2:**
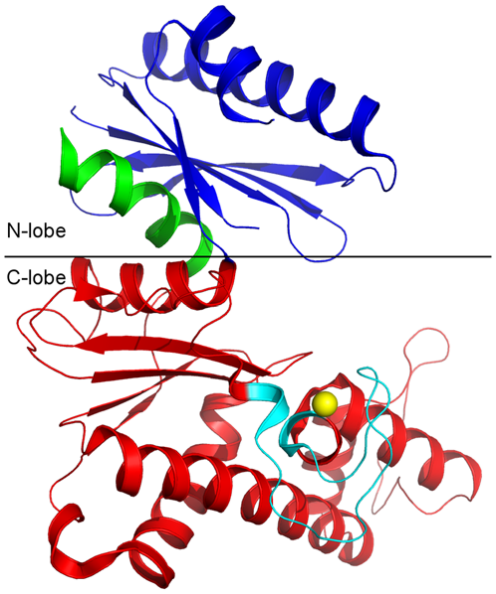
Overall structure of the kinase domain of GNE. The C-terminal helix (residues 700–717, in green) is embedded into the N-lobe subdomain. The ROK family signature zinc-binding motif (cyan) is located on top of two parallel helices of the C-lobe. Residues 475–498 in the N-lobe are missing in the electron density map and are presented by dotted line (black).

The overall structure adopts a typical bi-lobal kinase architecture. Both the N-lobe and the C-lobe have the α/β fold. Each lobe consists of a central β-sheet flanked by α-helices on both sides of the sheet. The last helix C-terminal to the C-lobe is part of the N-lobe and perpendicular to the interfacial helix of the C-lobe. Residues 475–498 of the N-lobe are invisible in the electron density map (Figure 2).

The GNE kinase domain contains a type I zinc-binding motif GHx_9–11_CxCGx_2_G(C/H)xE, which forms an HC3 type zinc-finger with residues H569, C579, C581, C586. The zinc-binding motif is a characteristic feature for all ROK family members [14]. The kinase domain also contains a DxGxT type ATP-binding motif [15], [16]. The side chains of this ATP-binding motif residues point toward the cleft between the N-lobe and the C-lobe. Comparison with the actin/hexokinase/hsp70 ATPase domains suggests that the disordered residues 475–498 form part of the binding pocket for the adenosine moiety of ATP [17] and are located near the DxGxT ATP-binding motif. Taken together, these findings suggest that the ATP binding pocket of the GNE kinase domain is located in the cleft between the two lobes.

### Oligomeric state of the GNE kinase domain

**Figure 3:**
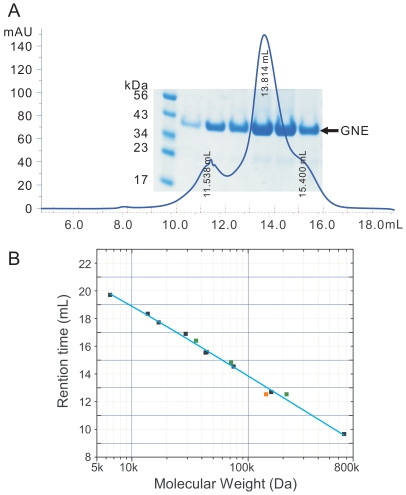
Oligomeric state analysis of the GNE kinase domain. Panel A: Superdex-200 10/300 (GE healthcare) size exclusion chromatography profile of GNE at 6 mg per mL concentration in 20 mM HEPES buffer, pH 7.5, 500 mM NaCl, 5 mM MgCl_2_, 1 mM tris-(2-carboxyethyl)phosphine hydrochloride (TCEP). The lanes on the SDS-PAGE gel correspond to different fractions on the gel filtration profile. Panel B: Size exclusion column calibration curve and measurement of GNE kinase domain apparent molecular weight. Standard protein samples (black data points) used include thyroglobulin (670 kDa), globulin (158 kDa), conalbumin (75 kDa), ovalbumin (44 kDa), anhydrase (29 kDa), myoglobin (17 kDa), and ribonuclease (13.7 kDa). Green data points are values for GNE kinase domain in monomeric, dimeric and hexameric forms. The orange data point is for GNE kinase domain in putative tetrameric form.

Previous deletion mutations study has suggested that the GNE kinase domain is responsible for dimerization, while a segment of residues between the epimerase and kinase domains, residues 360–382, is a potential site for trimerization [18]. Our gel filtration data (Figure 3) show that the kinase domain exists predominantly as a dimer in the solution, with small amounts of monomer and a higher order oligomer. The apparent molecular weight of the oligomer fits a hexamer of the kinase domain (see also below). However, the possibility of a tetramer [19] cannot be completely ruled out due to the low resolution of the gel-filtration column at this molecular size range (Figure 3b). The populations of the different oligomeric states of the protein are concentration dependant (data not shown), implying that the different oligomeric states exist in equilibrium.

**Figure 4:**
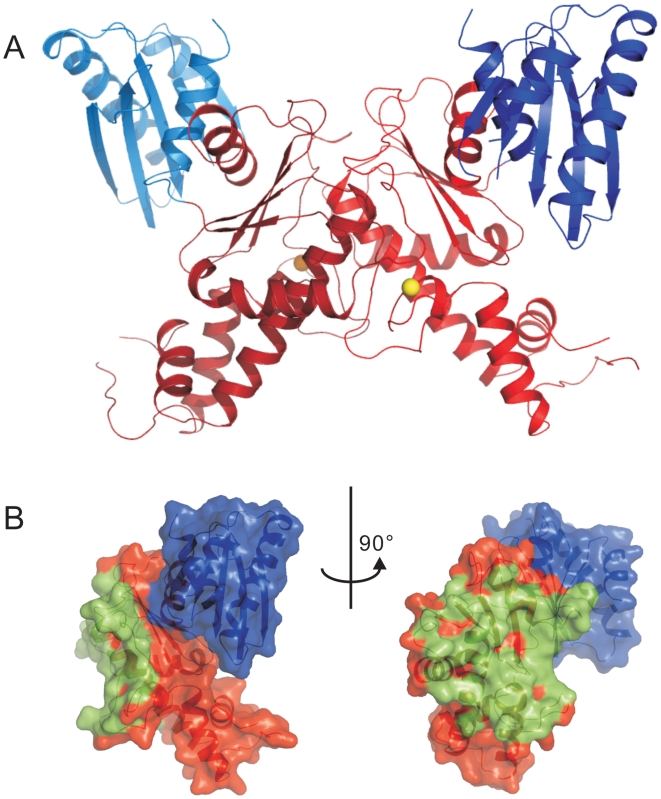
Dimeric GNE kinase domain. Panel A: Ribbon representation of the dimeric structure of the kinase domain. The N-lobes are shown in blue colors, the C-lobes in red. Panel B: Orthogonal views of the protein surface of one of the monomers. Residues within 4 Å distance from the other monomer are colored green.

**Figure 5:**
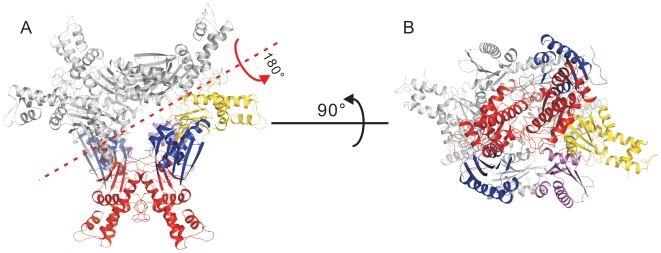
Crystallographic hexamer. Panel A: A crystallographic hexamer is produced by applying a two-fold rotational symmetry operation (red axis) on the three molecules in the asymmetric unit. The N-lobes of the dimer in the biological unit are shown in blue while the C-lobe in red. The N-lobe of the third molecule that forms a dimer with a symmetry-related molecule is shown in magenta, and the C-lobe in yellow. The three symmetry-related molecules are in grey. Panel B: A bottom-up view of the hexamer by rotating the hexamer in panel A over 90° along x-axis. The N-lobes of each dimer are located on the opposite sides of the hexamer.

The GNE kinase domain was crystallized in space group P3_1_21 with three molecules in the asymmetric unit. Protein interface and assembly analysis using the PISA server [20] suggests that two of the three molecules dimerize through the C-lobe with an average buried surface area of 1587 Å^2^ per molecule (Figure 4) whereas the third molecule dimerizes with a two-fold symmetry related molecule through the same C-lobe (Figure 5a). The solvation free energy gain upon formation of the interface, Δ^i^G, is −24.2 kcal·mol^−1^, indicating that the dimer interface is very stable and may not simply be a crystal packing artifact.

A crystallographic hexamer can be produced when a two-fold rotational symmetry operation is applied to the three molecules (one and a half dimers) in the asymmetric unit (Figure 5). In this hexamer, the N-lobes of three kinase molecules are pointing to the same side of the “hexamerization plane”, while the N-lobes of the other three molecules are pointing to the opposite side of the plane (Figure 5b). This assembly mode of the kinase domain allows locating the epimerase domain further away from the hexamerization plane and is consistent with the proposition that the interdomain segment (residues 360–382) is the site of trimerization [18].

### Structure comparison with other ROK family members

Structural homology search of the GNE kinase domain using the FATCAT (Flexible structure AlignmenT by Chaining Aligned fragment pairs allowing Twists) server [21] revealed the top four non-redundant hits to be PDB codes 2aa4, 1xc3, 1z05 and 1z6r. All these structures contain the signature zinc-binding motif of the ROK family. The structure of *E. coli* putative ManNAc kinase (PDB 2aa4) was the top hit with twist-adjusted r.m.s. deviation (opt-rmsd value) of 1.94 Å. The structure of a putative sugar kinase from *Bacillus subtilis* was the second best hit (PDB 1xc3). The other two homologous structures were transcription repressors that belong to the ROK family (PDB: 1z05 and 1z6r). *Vibrio cholerae* transcriptional regulator (PDB: 1z05) is a homolog of the *E. coli* Mlc protein (PDB: 1z6r). The latter is a transcriptional repressor that controls the expression of malT, the central transcription activator of the *E. coli* maltose system [22]. The structure of the GNE kinase domain aligns well with the *E. coli* Mlc structure: the N-lobe of GNE kinase domain aligns to the E-domain of Mlc and the C-lobe aligns to the O-domain of Mlc. It is interesting to note that the Mlc O-domain is responsible for the oligomerization of Mlc protein [22] in a way similar to the dimerization of GNE kinase through the C-lobe. However, these four structures do not contain sugar ligands that would help inform on a substrate binding mode for GNE.

**Figure 6:**
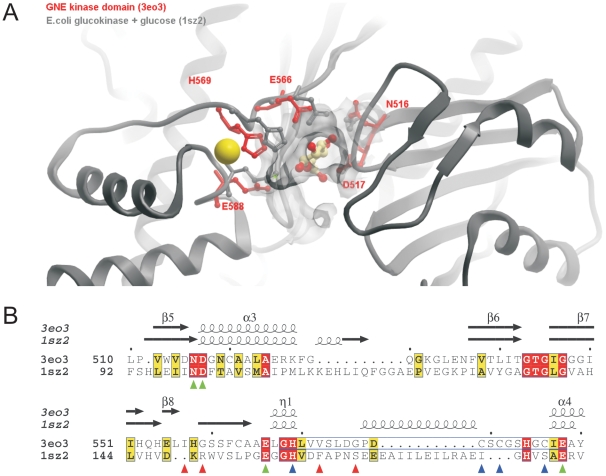
Putative sugar binding residues in the GNE kinase domain. Panel A: structural alignment of GNE kinase (red, 3eo3, only key residues putatively involved in sugar binding are shown as sticks) with *E. coli* glucokinase + glucose complex (grey, PDB 1sz2). Panel B: Structure-based alignment of the two proteins, only the segment involved in sugar binding is shown. The key residues are marked using green triangles. Residues involved in zinc-binding are marked using blue triangles. HIBM-related mutation sites are marked using red triangles.

To evaluate the putative sugar binding site, the sequence of the GNE kinase domain was aligned with that of *E. coli* glucokinase complexed with glucose (PDB: 1sz2) [23], which is the closest homologous structure containing a bound substrate currently available in the PDB data bank. The five residues involved in sugar binding are conserved in GNE (N516, D517, E566, H569, E588, GNE numbering). These five residues are arranged to accommodate the sugar substrate (Figure 6). Two residues, H569 and E588, are located in the ROK family zinc-binding signature motif and H569 directly coordinates the zinc ion. This finding suggests that zinc may play a catalytic role in sugar substrate binding, as well as a structural role.

### Structural mapping of disease-related GNE mutations

Since the identification of the relationship between *gne* mutations and HIBM [13], more than 60 mutations have been found to be associated with HIBM [24]. Among these mutations, 25 missense mutations at 23 unique sites are located in the kinase domain of the GNE protein. These 23 mutation sites can be classified into 4 different groups based on their solvent accessibility, and their locations (Table 1).

**Table 1:** Structural analysis of HIBM-associated missense mutations in the GNE kinase domain.

Missense Mutation^a^	Epimerase Activity (%)^b^	Kinase Activity (%)^b^	Oligomeric State^c^	Relative Accessibility in monomer^d^	Relative Accessibility in dimer^d^	Secondary structure element^e^	Mutation site feature	Mutation group
V421A				0.007	0.007	β-strand	Buried	IV
A460V				0.018	0.018	Helix	Buried	IV
I472T	50	5		0.000	0.000	β-strand	Buried	IV
P511H				0.416	0.416	Loop	Exposed	III
P511L				0.416	0.416	Loop	Exposed	III
N519S	40	20	hexameric	0.027	0.027	Helix	Interlobar	II
A524V	5	50		0.000	0.000	Helix	Interlobar	II
F528C	70	35	hexameric	0.140	0.140	helix end	Interlobar	II
F537I	45	60		0.000	0.000	β-strand start	Buried	IV
I557T				0.297	0.005	Loop	Dimer	I
G559R				0.470	0.229	Loop	Dimer	I
V572L	70	10		0.221	0.000	Loop	Dimer	I
G576E	15	15	trimeric	0.254	0.254	Loop	Dimer	I
I587T	55	35		0.000	0.000	helix start	Buried	IV
A591T				0.009	0.009	Helix	Buried	IV
A600T				0.000	0.000	Helix	Buried	IV
A630T	80	40		0.000	0.000	Helix	Buried	IV
A631T	80	75	hexameric	0.109	0.109	Helix	Buried	IV
A631V	70	65	hexameric	0.109	0.109	Helix	Buried	IV
I656N				0.038	0.005	Helix	Buried	IV
Y675H				0.000	0.000	Helix	Buried	IV
V679G				0.000	0.000	Helix	Buried	IV
V696M				0.020	0.020	β-strand	Buried	IV
G708S	45	5		0.000	0.000	β-strand	Interlobar	II
M712T	100	70		0.155	0.155	Helix	Interlobar	II

a Data of missense mutations were extracted from reference [11] and [24].

b UDP-GlcNAc epimerase and ManNAc kinase activities are percentage values relative to the corresponding activities of the full length wild type GNE. Data extracted from reference [11].

c Oligomeric state of the full length mutant GNE. Data extracted from reference [11].

d Relative solvent accessibility of the residue calculated using the DSSP program [30] and normalized according to values in reference [31]. A value of 1 means full exposure of the residue while a value of 0 means the residue is fully buried.

e The type of the secondary structure element the residue is located at was assigned using the DSSP program [30].

**Figure 7:**
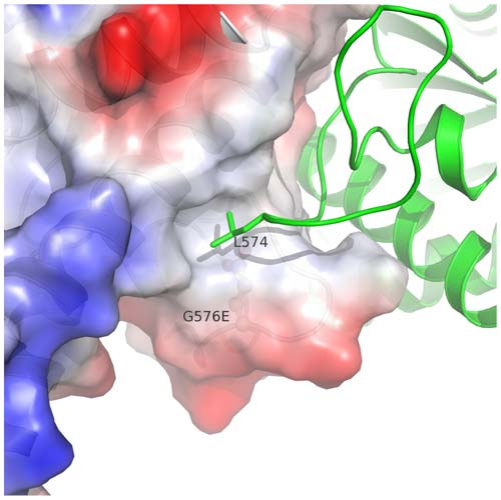
Dimer interface around residue G576. Surface charge of one molecule is shown around residue G576 (red for negatively charged surface, blue for positively charged surface). Side chain of L574 from another molecule is buried into the hydrophobic patch around G576. The side chain of the glycine residue is mutated to that of a glutamic acid residue and shown in ball-stick mode.

The first group of residues I557, G559, V572, and G576 is located at the dimerization interface of the C-lobe and mutation of these residues may interfere with dimerization of the kinase domain. It is noteworthy that kinase domain dimerization does not affect the solvent accessibility of G576 (Table 1), indicating that G576 is not directly involved in dimerization. The amino acid side chain of a mutant at this position would point into a hydrophobic niche that also accommodates the side chain of L574 from another monomer. The G576E mutation would exert both charge and space hindrances on the side chain of L574 and thus disrupt the dimerization (Figure 7), consistent with the previous observation that the G576E mutant of the full length GNE remains as a trimer [11].

**Figure 8:**
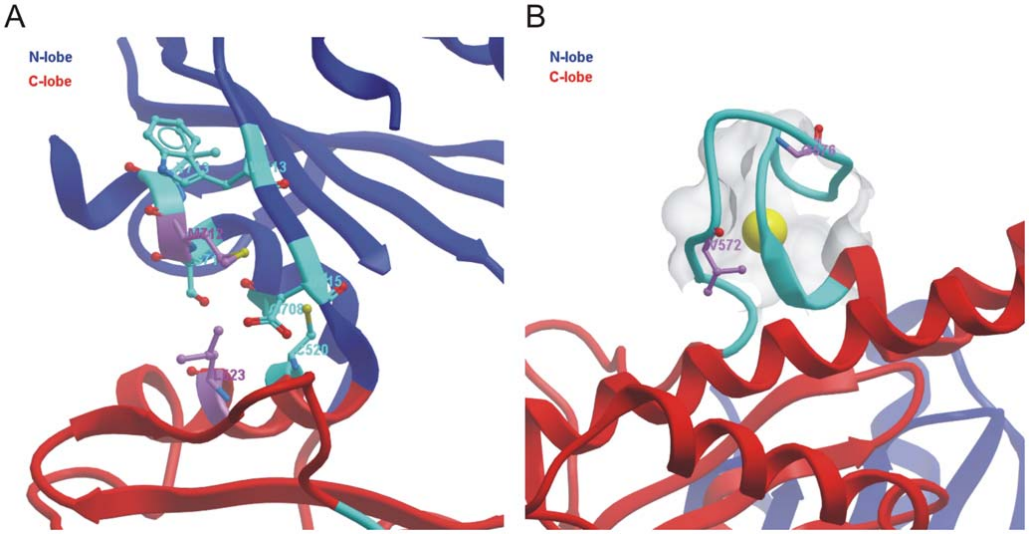
Disease-related mutations in the GNE kinase domain. Panel A: M712 and its surrounding environment. M712 is within 4 Å with the side chain of L523 and forms hydrophobic interaction. Panel B: Disease-related mutations at residues V572 and G576 are located in the zinc-binding motif.

The location of this group of residues is also close to the residues involved in sugar substrate binding (Figure 6b). Residues V572 and G576 are located on the zinc-binding signature motif of the ROK family (Figure 6b, 8b), which could play both a functional and a structural role. Mutations of these residues could thus also affect the sugar substrate binding affinity of the kinase domain indirectly.

The second group of residues includes those located at the interfacial helices between the N-lobe and the C-lobe, i.e. N519, A524, F528, G708, and M712. Since the interlobar cleft is the site of ATP and carbohydrate binding as well as where phosphoryl transfer occurs, mutation of these residues could change the interlobar movement during catalysis and thus affect the kinase activity of the protein. For example, the first identified HIBM-related mutation, M712T [13], would likely abolish the hydrophobic interaction of the side chain of M712 with that of L523 from the C-lobe helix (Figure 8a). In the previous study [11], the M712T mutation has been shown to cause a 30% reduction in the kinase activity without affecting the epimerase activity of full length GNE. On the contrary, mutations of other residues in this second group reduce not only the kinase activity but also the epimerase activity of the full length protein ([11], Table 1). This suggests that the kinase domain is allosterically coupled to the epimerase domain. The structure of the full length GNE is needed to fully understand the coupled effects of the kinase and epimerase domains.

The third group currently includes residue P511. P511 has the highest relative solvent accessibility (>40%) among the 23 mutation sites and is located on a loop region of the structure. The underlying mechanism for the association of P511H and P511L mutations with HIBM is elusive without further data, but mutation of a proline to any other residue type will inevitably change the flexibility of the loop region around this residue and could thus change the allostery of full length GNE in the higher-order oligomeric state.

The fourth group of residues includes all the rest of mutation sites in Table 1. All residues have hydrophobic side chains and low solvent accessibilities, and are located within secondary structural elements. Mutations of these residues may disrupt the secondary structural elements at given mutation sites, and could interfere with the hydrophobic interactions of the secondary structure elements that stabilize the protein quaternary structure.

### Summary

We show here the 3D structure of the N-acetylmannosamine kinase domain of GNE, the only ROK family kinase encoded in the human genome. The kinase domain dimerizes through an interface at the C-lobe. This is consistent with mutagenesis data from other groups on the full length GNE protein [11], [18]. The crystallographic hexamer, which consists of a trimer of kinase dimers, could serve as a prototype of a proposed full length GNE hexamer. Structure comparison of the GNE kinase domain with previously studied proteins revealed potential substrate binding sites at the interlobar cleft and also the structural and functional importance of the signature zinc-binding motif of the ROK family. Four groups of missense mutations associated with hereditary inclusion body myopathy are classified and their effects on the enzymatic activity can mostly be explained by the structure model.

## Materials and Methods

### DNA cloning, protein expression and purification

The cDNA template encoding the kinase domain of GNE was codon optimized for overexpression in *E. coli* and synthesized commercially (Codon Devices, Inc.) The DNA fragment encoding GNE residues 406–720 was PCR amplified and subcloned into the pET28-MHL vector (gi:134105571) using an In-Fusion dry-down PCR cloning kit (ClonTech). Protein was overexpressed in *E. coli* BL21(DE3) CodonPlus-RIL cells (Stratagene) grown in terrific broth medium. The culture was grown at 37°C in a LEX bubbling system (Harbinger Biotech. & Engineering Corp.) until OD_600_ reached 3.0. The temperature of the culture was then lowered to 15°C and the cells were induced with 0.5 mM isopropyl 1-thio-β-D-galactopyranoside and allowed to grow further overnight. Cells were harvested by centrifugation and flash frozen in liquid nitrogen and stored at −80°C until purification. Frozen cells were thawed and resuspended in 10 mM HEPES buffer (pH 7.5) containing 500 mM sodium chloride, 5% glycerol, 2 mM β-mercaptoethanol, and supplemented with 5 mM imidazole, and mechanically lysed using a microfluidizer (Microfluidics, model M-110EH) at 1,000 bar pressure. The lysate was clarified by centrifugation. GNE protein was bound with nickel-nitrilotriacetic acid (Ni-NTA) beads (Qiagen) at a ratio of 2.5 mL 50% Ni-NTA flurry per litre of cell culture. The bound protein was washed twice with the same HEPES buffer containing 30 mM or 75 mM imidazole, and finally eluted with the HEPES buffer supplemented with 300 mM imidazole. The elutant containing the GNE protein was further purified by Supderdex-75 size exclusion chromatography (GE Healthcare). The eluted fractions were pooled, concentrated to a final concentration of 40 mg per mL, and stored in a buffer containing 10 mM HEPES, pH 7.5, 500 mM sodium chloride, 5% glycerol and 5 mM dithiothreitol. The purity of the protein was better than 95% judging from SDS-PAGE gel.

Selenomethionine (SeMet) labelling of the protein was carried out using prepacked M9 SeMet growth media kit (Medicilon) following manufacturer's instructions.

### Crystallization and structure determination

The ligand-free form crystals were grown at room temperature in sitting drops. A final concentration of 5 mM ADP, 1∶100 chymotrypsin (w/w) were added into the protein stock solution and 0.5 µL protein solution was mixed immediately with 0.5 µL well solution containing 15% polyethylene glycol (PEG) 4000, 0.2 M ammonium acetate, 0.1 M sodium citrate, pH 5.6 and set up for vapour diffusion crystallization. The SeMet crystal used for structure determination was grown in 14.55% PEG4000, 0.2 M ammonium acetate, 0.1 M sodium citrate, pH 6.0, with 1∶100 chymotrypsin (w/w) and 5 mM ADP in a sitting drop setup. Crystals grew to a mountable size within 24 hours. Paratone oil was used to cryo-protect the crystals.

Diffraction data of a selenomethionyl derivative of the GNE kinase domain were collected at beamline 19ID of the Advanced Photon Source (APS) at a wavelength of 0.9792 Å. Initial phases were obtained by single wavelength anomalous diffraction with SOLVE and density modification with RESOLVE [25]. For model building, the phases were combined with data collected at APS beamline 23ID-B at a wavelength of 0.9793 Å (see Table 2). The refined model of the target resulted from iterative application of density modification with DM and RESOLVE, interactive model building with COOT [26], coordinate and B-factor refinement with REFMAC [27] and PHENIX [28], and geometry validation with MOLPROBITY [29]. Diffraction data and refinement statistics are summarized in Table 2. The current model was deposited at the Protein Data Bank with PDB ID 3EO3.

**Table 2:** Crystallographic data and refinement statistics.

	SeMet	Native
**Diffraction data** a		
Space group	P3_1_21	P3_1_21
Cell dimensions: a, c (Å)	128.88, 126.66	127.95, 127.25
Resolution (Å)	20.00−2.80 (2.90−2.80)b,c	30.00−2.84 (2.94−2.84)
Unique HKLs	27,466 (984)	29,072 (2,860)
Completeness (%)	90.7 (32.8)	100.0 (100.00)
*R_sym_* d (%)	14.7 (>1)	8.9 (97.8)
<*I/σI*>	20.5 (0.4)	40.0 (3.0)
Redundancy	8.1 (1.5)	11.1 (10.5)
**Refinement**		
Initial phasing mean FOM^e^	-	0.30
No. of atoms: protein/others	-	5,885/3
*R_work_*/*R_free_* f (%)	-	20.5/24.5
Coordinate error^g^ *R_work_*/*R_free_*/max likelihood (Å)	-	0.71/0.32/0.29
RMSD bond length (Å)/angle (°)	-	0.012/1.1
Mean B-factor (Å^2^)	-	73.8
Ramachandran plot favored/outliers (%)	-	96.1/0.0

a Data reduced with DENZO and SCALEPACK.

b Numbers in parentheses are for outer shell.

c Phasing data were complete only to 3.15 Å, but were not re-scaled to that resolution limit.

d *R_sym_* = *Σ|I-<I>|/ΣI*.

e FOM: Figures of merit.

f *R_work_* = *Σ||F_o_|−|F_c_||/Σ|F_o_|*, where *F_o_* and *F_c_* are the observed and calculated structure factors, respectively. *R_free_* was calculated as *R_work_* by using 3.8% of the data selected in thin resolution shells with SFTOOLS.

g Estimated standard coordinate uncertainty.

## Supporting Information

Datapack S1 Standalone iSee datapack - contains the enhanced version of this article for use offline. This file can be opened using free software available for download at http://www.molsoft.com/icm_browser.html.
(ICB)

Text S1 Instructions for installation and use of the required web plugin (to access the online enhanced version of this article).
(PDF)
